# Supplementary material for: Early detection of depression using a conversational AI bot: A non-clinical trial
Source: PLoS One. 2023 Feb 3;18(2):e0279743. doi: 10.1371/journal.pone.0279743 (PMC9897524; doi:10.1371/journal.pone.0279743)
Supplement: S1 Appendix — (PDF) [file pone.0279743.s001.pdf]

Appendix

Appendix A Full Conversation Design comprising 27 questions:
[Microsoft Power Point Presentation, 82KB-Multimedia Appendix A].

Appendix B Atomic Data of Focused Group Responses
[Microsoft Excel Worksheet, 53KB-Multimedia Appendix B].

Appendix C Consent Form for participants involved in research
[Microsoft Word Document, 46KB-Multimedia Appendix C].

Appendix D Ethics Application related to this study
[Microsoft Edge Pdf Document, 293KB-Multimedia Appendix D].

Appendix E User Satisfaction Form
[Google Forms, 98KB-Multimedia Appendix E].

Appendix F DEPRA Chatbot Scoring - 50 Participants
[Microsoft Excel Worksheet, 69KB-Multimedia Appendix F].
