# Supplementary material for: Early detection of depression using a conversational AI bot: A non-clinical trial
Source: PLoS One. 2023 Feb 3;18(2):e0279743. doi: 10.1371/journal.pone.0279743 (PMC9897524; doi:10.1371/journal.pone.0279743)
Supplement: S1 File — (DOCX) [file pone.0279743.s002.docx]

All repository links are embedded within the manuscript.

Here they are again for ready reference:

1. Full Conversation Design comprising 27 questions: https\%3A\%2F\%2Fraw.githubusercontent.com\%2FPayamK1\%2FHDRS_FlowChart\%2Fmain\%2FChatbot\%2520Conversational\%2520Flow_green.pptx&wdOrigin=BROWSELINK

2. Atomic Data of Focused Group Responses: https://view.officeapps.live.com/op/view.aspx?src=https\%3A\%2F\%2Fraw.githubusercontent.com\%2FPayamK1\%2FFocused-Gropu-Responses\%2Fmain\%2FFocused\%2520Group\%2520Responses.xlsx&wdOrigin=BROWSELINK

3. Consent Form for participants involved in research: https\%3A\%2F\%2Fraw.githubusercontent.com\%2FPayamK1\%2FConsent-Form\%2Fmain\%2FVU-HREApplication-Consent-Form-for-Participants-Involved-in-Research-Final.docx&wdOrigin=BROWSELINK

4. Ethics Application related to this study: https://github.com/PayamK1/Ethics-Application/blob/main/Ethics\%20Application.pdf

5. User Satisfaction Form: https://forms.gle/yk9BTM34d2d96WWd7

6. DEPRA Chatbot Scoring - 50 Participants: https://view.officeapps.live.com/op/view.aspx?src=https\%3A\%2F\%2Fraw.githubusercontent.com\%2FPayamK1\%2FDEPRA-Dataset-With-50-Participants\%2Fmain\%2FDEPRA\%2520Dataset_16\%2520Nov\%25202021_N\%253D50.xlsx&wdOrigin=BROWSELINK
