# Supplementary material for: Early detection of depression using a conversational AI bot: A non-clinical trial
Source: PLoS One. 2023 Feb 3;18(2):e0279743. doi: 10.1371/journal.pone.0279743 (PMC9897524; doi:10.1371/journal.pone.0279743)
Supplement: Attachment — Submitted filename: Response to Reviewers PONE-D-22-04147.pdf [file pone.0279743.s005.pdf]

Response Letter

Date: 24th November 2022

Dear Reviewers,

We appreciate your valuable comments and revisions of the submitted manuscript on behalf of the authors at Victoria University Footscray Park in Melbourne, Australia. The manuscript entitled "Early Detection of Depression Using Conversational AI Bot: A Non-Clinical Trial", with manuscript number PONE-D-22-04147.

We have reviewed and applied the comments carefully with attention to detail. The manuscript has been updated with modifications marked in **blue (first round)**, **purple (second round)** and **red (third round)** throughout the edited version. We hope that the revised version will meet the criteria commented on by the reviewers and meet the journal's standards. The following pages provide explanations for each comment.

If you would like to discuss any comments below, please do not hesitate to contact the authors.

Best regards,

Khandakar Ahmed

Email: khandakar.ahmed@vu.edu.au

All authors list:

1. Payam Kaywan, Victoria University, Victoria, Melbourne, Australia
2. Khandakar Ahmed, Victoria University, Victoria, Melbourne, Australia
3. Ayman Ibaida, Victoria University, Victoria, Melbourne, Australia
4. Yuan Miao, Victoria University, Victoria, Melbourne, Australia
5. Bruce Gu, Victoria University, Victoria, Melbourne, Australia

Third Round Review Response

Response to Academic Editor's Comments

Manuscript No. PONE-D-22-04147
Manuscript Title: "Early Detection of Depression Using a Conversational AI Bot: a Non-Clinical Trial"

Message to Reviewers and Academic Editors: On behalf of the authors' team, we would appreciate your time and consideration in reviewing this manuscript. In the following section, we have addressed the points and hints from your point of view. We have tried our best to discuss the matters with attention to detail and make the manuscript more practical and meaningful.

Responses to Academic Editor's Comments

Comment 1) I have one concern regarding the paper. The ethical considerations are not discussed by the authors in the manuscript. Have they followed proper ethical guidelines for conducting this research?

Response:

The manuscript discusses the ethical consideration in detail on page#13 as a separate sub-section. The authors also included the approved complete ethics application and consent form in the Appendix.

For your ready reference, here is the excerpt or summary from the section

This research is registered under ethics approval number HRE 20-184 at Victoria University, Melbourne, Australia. It involves a non-clinical population of participants who reside in Australia at the time of the research. The participants are between 18 and 80 years old and willing to reveal information on their moods [42] and emotions [43] in a scientific trial. **Appendix C** provides the consent form for the participants involved in the research, and **Appendix D** provides the Ethics Application related to this study.

The participants indicated their willingness to interact with the DEPRA chatbot and participate in the study by signing the consent form electronically. The participants were prompted to access the consent form after contacting DEPRA. All participants must agree to the terms and conditions in the consent form, which notes that DEPRA may record and analyse their responses to the questions relating to moods, sleep habits, appetite, general health symptoms, etc.

This research deals with human beings and their moods and behaviours in general. The ethics committee follows strict guidelines and conditions to confirm the validity of the methods and approach used by the research team to conduct the research. One of the ethics committee's main concerns is how the study maintains the participants' privacy. After completion of the data collection by the research team, the data is stored on an R drive and a computer with limited

access. Only the research team members are allowed access to the raw data. The participants are assured that no unauthorised users will access the raw data. All data were de-identified to protect the privacy of the participants.

Besides this dedicated section, the authors also discuss the ethical considerations on page#12 under the subsection '**Participant Recruitment**' and page#18~19 under '**Discussions and Future Work.**'
